# Evaluation of a long day care intervention targeting the mealtime environment and curriculum to increase children’s vegetable intake: a cluster randomised controlled trial using the multiphase optimisation strategy framework

**DOI:** 10.1017/S1368980024000557

**Published:** 2024-02-26

**Authors:** Samantha Morgillo, Lucinda K Bell, Claire Gardner, Shabnam Kashef, Karen Stafford, Dorota Zarnowiecki, Astrid AM Poelman, Maeva O Cochet-Broch, Brittany J Johnson, Aarti Gulyani, David N Cox, Rebecca K Golley

**Affiliations:** 1 Caring Futures Institute, College of Nursing and Health Sciences, Flinders University, GPO Box 2100, Bedford Park, SA, Australia; 2 Nutrition Australia Victorian Division, Carlton, VIC, Australia; 3 Commonwealth Scientific and Industrial Research Organisation (CSIRO), Health and Biosecurity, Westmead, NSW, Australia; 4 Commonwealth Scientific and Industrial Research Organisation (CSIRO), Agriculture & Food, North Ryde, NSW, Australia; 5 Commonwealth Scientific and Industrial Research Organisation (CSIRO), Health and Biosecurity, Adelaide, SA, Australia; 6 Research and Innovation Services, University of South Australia, Mawson Lakes, SA, Australia

**Keywords:** Children, Vegetable intake, Long day care, Multiphase optimisation strategy, Randomised controlled trial

## Abstract

**Objective:**

To determine the reach, adoption, implementation and effectiveness of an intervention to increase children’s vegetable intake in long day care (LDC).

**Design:**

A 12-week pragmatic cluster randomised controlled trial, informed by the multiphase optimisation strategy (MOST), targeting the mealtime environment and curriculum. Children’s vegetable intake and variety was measured at follow-up using a modified Short Food Survey for early childhood education and care and analysed using a two-part mixed model for non-vegetable and vegetable consumers. Outcome measures were based on the RE-AIM framework.

**Setting:**

Australian LDC centres.

**Participants:**

Thirty-nine centres, 120 educators and 719 children at follow-up.

**Results:**

There was no difference between intervention and waitlist control groups in the likelihood of consuming any vegetables when compared with non-vegetable consumers for intake (OR = 0·70, (95 % CI 0·34–1·43), *P* = 0·32) or variety (OR = 0·73 (95 % CI 0·40–1·32), *P* = 0·29). Among vegetable consumers (*n* 652), there was no difference between groups in vegetable variety (exp(b): 1·07 (95 % CI:0·88–1·32, *P* = 0·49) or vegetable intake (exp(b): 1·06 (95 % CI: 0·78, 1·43)), *P* = 0·71) with an average of 1·51 (95 % CI 1·20–1·82) and 1·40 (95 % CI 1·08–1·72) serves of vegetables per day in the intervention and control group, respectively. Intervention educators reported higher skills for promoting vegetables at mealtimes, and knowledge and skills for teaching the curriculum, than control (all *P* < 0·001). Intervention fidelity was moderate (*n* 16/20 and *n* 15/16 centres used the Mealtime environment and Curriculum, respectively) with good acceptability among educators. The intervention reached 307/8556 centres nationally and was adopted by 22 % eligible centres.

**Conclusions:**

The pragmatic self-delivered online intervention positively impacted educator’s knowledge and skills and was considered acceptable and feasible. Intervention adaptations, using the MOST cyclic approach, could improve intervention impact on children’ vegetable intake.

Globally children are not consuming enough vegetables^(1,2)^ with < 1 % of children in Australia aged 2–8 years meeting national recommendations for vegetable consumption^(3)^. Poor consumption of vegetables in childhood can have negative implications on growth, health and development^(4,5)^. Intervening to improve vegetable intake early in life in settings where children spend their time is therefore needed.

Early childhood education and care (ECEC) services are a promising setting for intervention due to the high proportion of preschool-aged children (87 % in high-income countries^(6)^) attending ECEC. In Australia, over half of children aged under 5 years attend ECEC services for an average of 30 hours per week^(7)^. The most common ECEC service in Australia is long day care (LDC)^(8)^, where centre-provided meals can contribute up to two-thirds of children’s daily intake^(9,10)^. Concerningly, children do not consume the recommended amounts of vegetables in care^(11,12)^. Previous interventions in the ECEC setting have demonstrated capacity to improve children’s dietary intake^(13–16)^; however, few (*n* 16/55) have investigated the specific impact on vegetable intake^(14)^. Thus, while LDC is an opportunistic setting to influence children’s eating behaviour, a gap remains regarding effective interventions to improve children’s vegetable intake.

Interventions that have improved children’s food intake in ECEC settings have commonly been multi-component, targeting both environmental (i.e. centre-level food service, educators’ feeding practices) and individual (i.e. children by the curriculums) level determinants^(13,14)^. However, delivering and evaluating multi-component interventions using a traditional randomised controlled trial design can present challenges for scalability due to the controlled conditions not being representative of real-world conditions^(17)^. In addition, multiple resource intensive and costly trials are required to evaluate the effectiveness of each component. To overcome such challenges, the multiphase optimisation strategy (MOST) can be used to efficiently develop and evaluate effective and scalable behavioural multi-component interventions^(18)^. The MOST framework uses a cyclic approach and comprises three phases: (1) Preparation, (2) Optimisation and (3) Evaluation^(18)^. Using this framework, we previously developed three initiatives to increase children’s vegetable intake in LDC: (1) Food provision, (2) Mealtime environment and (3) Curriculum^(19)^ (preparation phase) and tested them using an eight-group factorial trial to determine the most efficient intervention (optimisation phase)^(20)^. We found that the Mealtime environment with Curriculum initiative combination had the most promise for increasing children’s vegetable intake^(20)^. Using a weighed plate waste method, a non-significant and clinically meaningful increase of 0·36 serves of vegetables per day was observed compared with the control group^(20)^. Initiative fidelity was also greatest for the *Curriculum* initiative (>90 % completion rates), and good acceptability was reported for both the *Curriculum and Mealtime environment initiatives* (4/5 educators would recommend to others). Evaluating this optimised intervention in a randomised controlled trial is the next step of the MOST framework.

Thus, the current paper reports on the evaluation phase of the MOST framework to determine the effectiveness of the optimised intervention (Mealtime environment with Curriculum initiatives)^(20)^ on children’s vegetable intake in LDC. The RE-AIM framework has been employed to understand the potential of the intervention to be upscaled into LDC settings nationally^(21)^. The objectives were to evaluate the reach, adoption, implementation and effectiveness of the intervention on increasing children’s vegetable intake while in LDC. The primary outcome was children’s vegetable intake (effectiveness), and it was hypothesised that children in the intervention group would consume 0·5 serves more vegetables per day than children in the waitlist control group. The effect of the optimised intervention on the secondary outcomes of educators’ knowledge and skills (effectiveness), intervention fidelity and acceptability (implementation), reach and adoption were also assessed.

## Methods

### Study design

This study was a cluster randomised controlled trial to determine the effectiveness of a 12-week intervention targeting the Mealtime environment and Curriculum on children’s usual vegetable intake in LDC. The pragmatic intervention was provided online and self-delivered by centres to replicate real-world conditions. The trial was prospectively registered with the Australia and New Zealand Clinical Trials Registry (ACTRN12620001323910p) and follows the consolidated standards of reporting trials reporting checklist for cluster randomised controlled trials. Ethics approval was obtained from the Flinders University Human Research Ethics Committee (project number #4764).

### Sample and recruitment

Private (non-government) LDC were approached to participate in this study. Government LDC were not approached due to the different ethics approval processes required. Centres were eligible to participate if they were: (1) LDC centres located in Australia who operate for at least 8 hours per weekday (Monday to Friday), (2) care for children aged 2–5 years, (3) are responsible for the centre menu planning and (4) prepare food onsite serving lunch and two between-meal snacks each day (morning and afternoon). Centres were excluded if they catered exclusively to children with special needs or if they participated in the previous optimisation study^(20)^. Within centres, children aged 2–5 years were eligible. Based on a 15 % attrition rate from prior research^(22)^, a total sample size of 392 children (30–40 centres) was required for 80 % power with a two-sided alpha of 0·05, calculated assuming a mean difference between the groups of 20 g ± 100 g (0·27 serves±1·33 serves) per day from prior research^(22)^. An intraclass correlation of 0·1 within each centre for vegetable intake was assumed based on prior research^(22,23)^.

An online expression of interest form, replicating real-world dissemination and comprising the eligibility assessment, was sent via email to a list of accredited LDC centres in Australia (*n* 5436), obtained from the Australian Children’s Education and Care Quality Authority website^(24)^ between February and April 2022. Large-chain LDC providers (*n* 3) with known contact details were encouraged to promote the study amongst their centres, a previously effective recruitment strategy^(25)^. Secondary recruitment methods included promotion using social media and newsletters through relevant stakeholder organisations (e.g. Early Childhood Australia and Australian Childcare Alliance). Centres that did not express interest in the study were followed up via email a maximum of two times. Eligible centres that completed the expression of interest form were invited to complete an online registration form which included the study information sheet, consent form and centre demographic questionnaire. Eligible centres that did not complete the registration form were followed up via email and/or phone a maximum of 1–2 times. Centre management (e.g. directors, nominated supervisors) was instructed to discuss participation with centre staff before providing consent and registering their centre in the study. Following registration, centres were instructed to distribute the online study information sheet along with the consent form to their educators. Centres were also instructed to distribute a parent information sheet including an opt-out consent form to families of enrolled children, allowing caregivers the opportunity to opt out of the study on behalf of their child. Therefore, all children whose caregivers did not opt-out were included.

### Randomisation and blinding

Registered centres were allocated to the control or intervention group using the randomisation module of Research Electronic Data Capture (REDCap), a secure, web-based software platform hosted by Flinders University and designed to support data capture^(26,27)^. Cluster randomisation was employed, in which children and educators attending the same centre were assigned the same intervention, using a block size of four generated in GraphPad (GraphPad Software, Inc). It was not possible to blind centres, families or research staff to group allocation at follow-up.

### The intervention

The intervention consisted of two initiatives targeting the Mealtime environment and the Curriculum^(19)^, developed based on the Best Practice Guidelines for Increasing Children’s Vegetable Intake in the Early Years^(28)^ and draw on evidence for effective strategies for increasing vegetable intake and acceptance in the early years from a recent umbrella review^(13)^. The intervention was delivered online whereby at the beginning of the 12-week study period centre management were provided with a website link and instructions for accessing the initiatives via email. Centres were expected to subsequently access and flexibly deliver the intervention to replicate real-world settings. The Mealtime environment initiative involved a 45–55-minute online training module for educators covering evidence-based feeding practices to promote vegetable acceptance, liking and intake during mealtimes (https://heas.health.vic.gov.au/training/training-early-childhood-sector). The Curriculum initiative provided centres with access to the Taste & Learn^TM^ for Early Years Curriculum guide^(29)^ which contained implementation advice and content for 16 × 20 min lessons accompanied with 10 min snack time activities (i.e. 2× lessons + 2× snack time activity per week) based on experiential learning, sensory education and vegetable preference development in children. More information describing the initiatives are available in the protocol^(19)^ and optimisation phase^(20)^ papers.

The 12-week study period comprised of a 4-week preparation phase to allow for participating educators to complete the training and plan for the Curriculum (e.g. source vegetables for the snack time activities), followed by an 8-week implementation phase to apply the learned feeding practices during mealtimes and deliver the curriculum in rooms with children aged 2–5 years.

Centres allocated to the waitlist control group were instructed to continue with their usual practice. Educators were instructed to not complete any nutrition training (excluding allergy and food safety training) and to not use any vegetable or nutrition curriculum at their centre during the 12-week study period. Waitlist control centres were provided with the intervention materials following completion of follow-up data collection at their centre.

### Data collection

All outcome data for this study were collected following the 12-week study period between May and July 2022 using REDCap online questionnaires^(26,27)^. In contrast to the protocol^(19)^, baseline data were not collected in this study as findings from the optimisation phase^(20)^ demonstrated a high turnover of children from baseline to follow-up, limiting paired data analysis. Further, to minimise the burden of data collection on participants, and with the aim of increasing compliance at follow-up, the pragmatic decision was made to only collect data at follow-up. The primary outcomes were children’s usual vegetable intake, including variety, and the secondary outcomes were educator knowledge and skills, intervention fidelity and acceptability and reach and adoption. Only outcomes related to the Mealtime environment and Curriculum initiatives were measured (e.g. data on centre cooks and menus were not collected given the Food provision initiative was not included in the evaluation phase study). All centres were contacted via email and phone call approximately 2 weeks before the data collection period (i.e. week 10) began. Immediately after completing the 12-week study period, all centre managers and educators were emailed links to the follow-up questionnaires with subsequent weekly reminder emails and phone calls over a 2-week period (if not completed). Incomplete questionnaires (i.e. those with significant missing responses) were followed up via email. Centres that did not provide any data during the data collection period were classified as lost to follow-up.

#### Demographic characteristics

Demographic characteristics were collected for centres, educators and children. Centre characteristics, collected at registration, included: centre location, number of enrolments, number of children identifying as Aboriginal and/or Torres Strait Islander, number of staff and the nutrition policies, menu guidelines and resources used at the centre. The number of enrolments was used to define the centre size as either small (≤50 children) or large (>50 children). The socio-economic position of centres was determined by applying the Index of Relative Socio-economic Disadvantage from the Australian Bureau of Statistics Socio-Economic Indexes for Australia (SEIFA) 2016 ranking within Australia^(30)^ to centre postal code and categorising into low (deciles 1–5) and high (deciles 6–10). Demographic characteristics of participating educators were collected at follow-up via questionnaire and included age, gender, qualifications and relevant experience. Child demographic characteristics were reported at follow-up by educators and included: age, gender, the number of days the child attends care and meals eaten while in care.

#### Primary outcomes

##### Effectiveness: children’s vegetable intake and variety

To determine effectiveness of the intervention, children’s usual vegetable intake was measured using a modified version of the educator-completed Short Food Survey for ECEC (SFS-ECEC)^(31)^. The SFS-ECEC was chosen as it can be completed by centre educators using an online data collection form making it an appropriate tool for real-world measurement. The SFS-ECEC captures an individual child’s (2–5 years) food group intake over the past month while in care as described by the Australian Guide to Healthy Eating^(32)^. It has been shown to be acceptable by educators in this setting, with appropriate validity for estimating intake at the group level^(31)^. Questions not relating to vegetable intake (i.e. questions pertaining to all other food groups including fruit, breads and cereals, dairy and dairy alternatives, meat and alternatives as well as discretionary choices) were removed, reducing the forty-seven-item questionnaire to seven items. Of these, six measured the frequency (times per day/week or doesn’t eat) and usual portion size (½ portion, 1 portion, 2 portions or doesn’t eat) for cooked vegetables (e.g. cooked green beans or cooked lentils), salad vegetables (e.g raw tomato, cucumber) and starchy vegetables (e.g. potato) as defined by the Australian Guide to Healthy Eating^(32)^. One open text item measured the variety of vegetables, where educators were asked *‘Think back to the two most recent days the child has attended the service: How many different types of vegetables did he/she eat?’.*

Instructions and supporting resources (a completed example of the modified SFS-ECEC and images illustrating example portion sizes, e.g. 1 portion = 1/4 cup of cooked broccoli or 3 florets) were provided to centres prior to follow-up data collection. Centres were instructed to complete the modified SFS-ECEC for children (whose caregiver had not provided opt-out consent) aged 2–5 years in two (where possible) participating rooms (e.g. toddler and kindy rooms) for each child in attendance on the day of the week with the highest attendance. To limit attrition due to the burden of educator-completed data collection, the protocol was amended whereby centres that expressed limited capacity to complete data collection due to limited staff resourcing/time, or COVID-19 pandemic impacts, for example, were instead instructed to complete the modified SFS-ECEC for at least ten children as opposed to every child in attendance.

The total portions per day of cooked, salad and starchy vegetables consumed were calculated using either ((frequency per day) × (usual portion size) or ((frequency per week × usual portion size)/days attending care). These were summed to determine total vegetable intake in portions per day, which was converted to serves according to the Australian Guide to Healthy Eating^(32)^ where ((two portions) = (one serve)). Vegetable variety data were reported as the number of types of vegetables consumed.

#### Secondary outcomes

##### Effectiveness: educator knowledge and skills

The secondary outcomes of educator knowledge and skills were assessed at follow-up using a previously developed Theoretical Domains Framework (TDF) questionnaire for cooks in LDC^(33)^. This questionnaire was adapted to be suitable for use with educators to evaluate the Mealtime environment and Curriculum initiatives and was piloted in the optimisation study^(20)^. These domains were chosen for this study as key implementation measures to help understand the feasibility of the intervention. The domains assessed educator’s knowledge and skills, with statements rated using a five-point Likert scale (from 1=strongly disagree to 5=strongly agree). The overall score for each domain was determined by summing the response scores and dividing by the number of responses^(34–36)^.

##### Implementation: fidelity and acceptability

The secondary outcomes of intervention fidelity and acceptability were assessed at follow-up using a questionnaire completed by the educator/s responsible for implementation of the initiatives. Fidelity was assessed according to the frequency of centres and educators who completed the online Mealtime environment training (i.e. Mealtime environment initiative) and delivered the lesson and snack time activities (i.e. Curriculum initiative). The fidelity for curriculum lessons and activities were assessed using a five-point Likert scale: none, some (*n* 1–7 lessons or activities), half (*n* 8), most (*n* 9–15) or all (*n* 16). Acceptability was assessed using a purposefully designed set of evaluation questions based on the Learning Object Review Instrument^(37)^, with twenty-one items covering the domains of *content quality, learning goal alignment, motivation, reusability/accessibility* and *duration* and statements rated using a five-point Likert scale (from 1=strongly disagree to 5=strongly agree).

##### Reach and adoption

To understand reach of the intervention using real-world dissemination strategies, the number of centres who expressed interest to participate in the study was compared with the target population of LDC centres in Australia^(38)^. To understand adoption at the setting level, the participation rate was defined as the number of centres that enrolled in the study compared with eligible centres who expressed interest to participate in the intervention but did not enrol. Reasons for non-participation were collected over phone/email and documented in an excel spreadsheet.

Centre characteristics of SEIFA (high/low) and state representation (Collected from the expressions of interest) of those centres who participated was compared with those that did not participate. The state representation of the study sample was compared with national data of LDC in Australia^(38)^.

##### Contamination

Contamination was measured at follow-up via open-ended questions (see online supplementary material, Supplementary File 2) that asked the centre director and educators to report if they had undertaken any nutrition or food-related training, used any additional nutrition or vegetable curriculums or changed their menus (director only) at any point during the study period.

### Statistical analysis

Analyses were conducted using IBM SPSS version 27 and STATA version 17.0 se using intention to treat analysis. All data were checked and cleaned prior to analysis. Data were assessed for normality, and non-normal (discrete) data were presented as median (IQR). Demographic data for the centres, educators and children are reported as frequency (%) or median (IQR). Centre characteristics (size and SEIFA grouping) were compared between centres who completed follow-up data collection and centres who withdrew or were lost to follow-up using *χ* ^2^ analyses.

The primary outcome of vegetable intake was reported for usual serves of vegetables per day and number of types of vegetables consumed (i.e. variety). The data for usual vegetable intake (serves per day) did not fit the planned linear mixed model^(19)^ as it was zero inflated (i.e. contains a substantial proportion of zero outcome values) and right skewed (i.e. data positively inclined towards right). Thus, a mixed effect two-part analysis was conducted in STATA 17.0, in which zero outcome values (non-vegetable consumers) and non-zero outcome values (vegetable consumers) were treated separately^(39)^. The models were adjusted for clustering as a random effect. A two-part model combines two distributions: Part 1 employed a mixed effect logistic regression model and the fixed effects determined the difference between intervention and control groups in whether children consumed or did not consume any vegetables. Part 2 employed a mixed Gamma regression model with only those children who consumed vegetables (>0 serves) to measure how much they consumed and determine the difference between the groups. The within-centre correlation and level of variability were accounted for as the random effect.

Similarly, vegetable variety (count data) was analysed using a mixed effect zero-inflated Poisson model. Part 1 determined whether children had consumed any variety of vegetables or none using a mixed logistic model, and part 2 measured the variety of vegetable intake (if > 0 vegetable variety) between the control and intervention group using a mixed zero-inflated Poisson model. All models used follow-up data and were adjusted for covariates of child age, child gender, centre size and centre SEIFA group. All models were tested for goodness of fit and a likelihood ratio (LR) test was conducted to test random effect variance, i.e. testing the mixed model (fixed and random effect model) *v*. simple regression model (fixed effect only).

Secondary outcomes of educator knowledge and skills were reported as median (IQR) for the overall score for the TDF domains. The distribution of overall scores for knowledge and skills was compared between groups using the Mann–Whitney *U* test. Intervention fidelity was reported as the frequency of centres (with at least one educator who used the initiatives) and educators who used the initiatives, and acceptability was reported as the number of educators who agreed or strongly agreed with the Learning Object Review Instrument framework statements. Reach was reported as the total number of centres that expressed interest over the total number of LDC centres in Australia according to national data from the Australian Children’s Education and Care Quality Authority report^(38)^. Adoption was reported as the total number of centres who enrolled to participate in the study compared with non-participating centres. The characteristics (SEIFA group and state representation) of centres that participated were compared with centres that did not participate using *χ* ^2^ analysis. Significance was considered at p < 0·05.

## Results

### Demographic characteristics

**Fig. 1:**
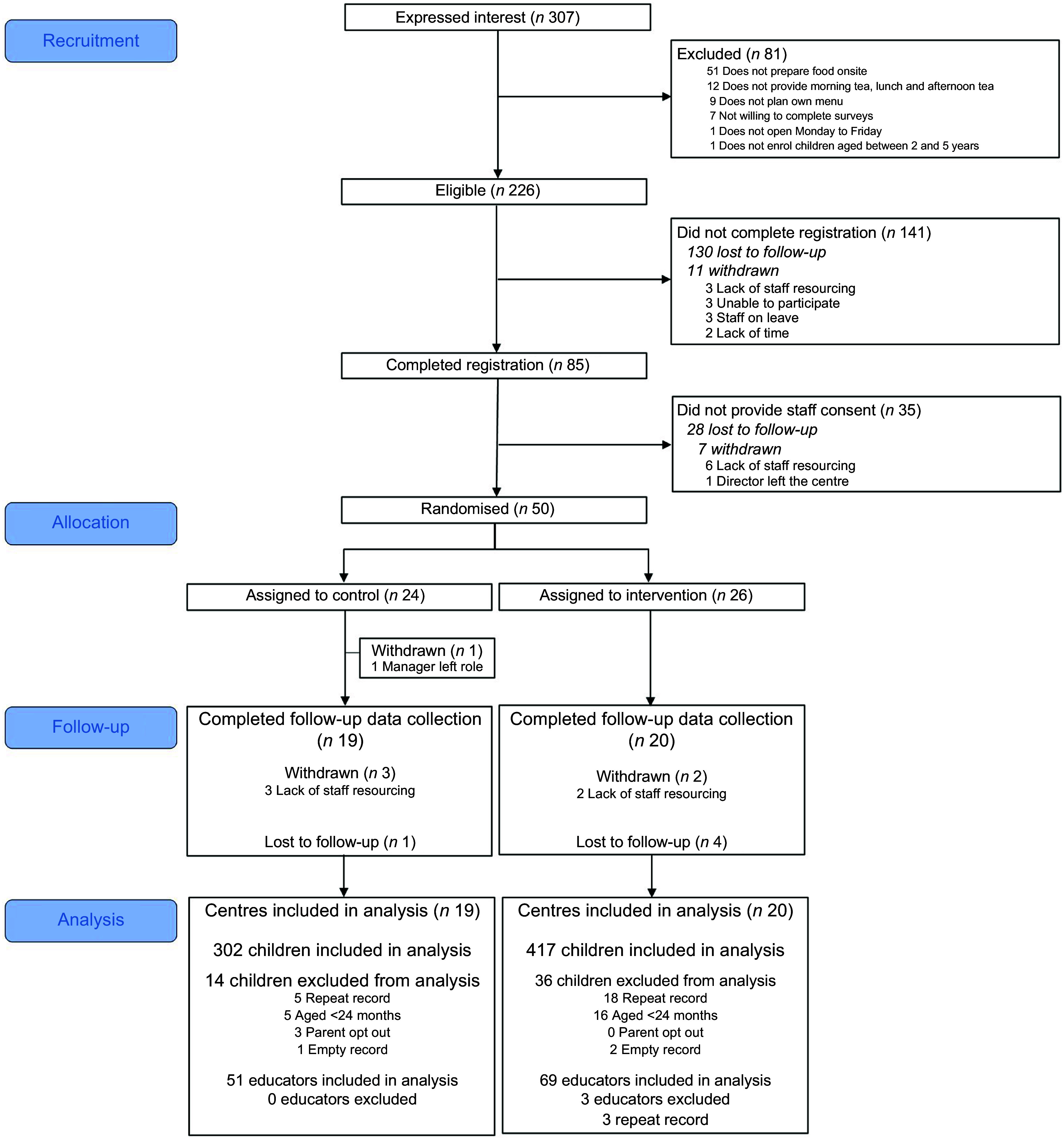
Flow diagram of centres according to the CONsolidated Standards of Reporting Trials (CONSORT) checklist

Figure 1 shows the flow of centres, educators and children through the study. Of the fifty centres that enrolled in the study (*n* 26 intervention, *n* 24 control), a total of thirty-nine centres completed follow-up data collection. One centre withdrew during the intervention period as a result of their manager leaving their role. During the follow-up data collection period, five centres withdrew due to lack of staff resourcing and five were lost to follow-up, due to not providing any completed questionnaires. There were no significant differences in socio-economic position (high/low SEIFA) (*P* = 0·38) or centre size (small/large) (*P* = 0·94) between those centres who completed data collection (*n* 39) and those that did not (*n* 11).

Characteristics of the sample at follow-up are presented in Table 1. Intervention centres comprised of centers in both low (*n* 10) and high (*n* 10) SEIFA areas across all states and territories in Australia other than Tasmania. Educators (*n* 120) were predominately female (95 %) with 8 years’ experience in the childcare setting (range, 3·0–15·0 years). Children (*n* 719 aged 3·7 (3·1–4·5) years) attended LDC for 3 (3–5) days per week on average and consumed 3 (3–4) meals per day in care.

**Table 1:** Characteristics for centres (*n* 39), children (*n* 719) and educators (*n* 120) presented as *n* (%) or median (IQR)

Characteristic	Control		Intervention		Total	
Centre characteristics	*n* 19	Median, IQR	*n* 20	Median, IQR	*n* 39	Median, IQR
**State**						
New South Wales	9	47	12	60	21	54
Queensland	4	21	1	5	5	13
Victoria	2	11	2	10	4	10
Western Australia	3	16	1	5	4	10
Australian capital territory	1	5	2	10	3	8
Northern territory	0	0	1	5	1	3
South Australia	0	0	1	5	1	3
Tasmania	0	0	0	0	0	0
**SEIFA group**						
Low (1–5)	10	53	10	50	20	51
High (6–10)	9	47	10	50	19	49
**Centre size**						
Small (<50 places)	9	47	9	45	18	36
Large (>50 places)	10	53	11	55	21	54
**No. children enrolled at the centre** (median, IQR)	56	43–90	55	44–74	56	44–79
**No. children identifying as aboriginal and/or torres strait islander** (median, IQR)	2	0–7	2	0–4	2	0–5
**No. cooks employed** (median, IQR)	1	1–1	1	1–1	1	1–1
**Centres with kitchen assistants**	3	16	3	15	6	15
**No. educators employed** (median, IQR)						
Certificate level	7	3–14	7	4–15	7	4–14
Bachelor level	9	5–17	7	3–10	7	4–12
**Nutrition policies and menu planning guidelines/nutrition resources used at the centre**						
Centre nutrition policy	19	100	20	100	39	100
Menu guideline	18	95	19	95	37	95
Start right-eat right (previous state government initiative)	3	16	0	0	3	8
Feed Australia	2	11	4	20	6	15
Healthy eating advisory service (nutrition Australia)	0	0	2	0	2	5
Nutrition Australia	9	47	8	40	17	44
Caring for kids	4	21	3	15	7	18
Get up and grow (commonwealth government guidelines)	6	32	6	30	12	31
Munch and move (NSW state government initiative)	9	47	14	70	23	59
Australian dietary guidelines (commonwealth government guidelines)	14	74	12	60	26	67
**Child sample characteristics**	***n*** **302**		***n*** **417**		***n*** **719**	
**Age (months)** (median, IQR)*	44·3	34·6–52·5	45·1	36·0–54·5	44·8	35·3–53·8
**Gender**						
Girl/female	145	48	190	46	335	47
Not specified	0		1	<1	1	<1
**Number of days attending care per week** (median, IQR)	4	3–4	3	3–5	3	3–5
**Number of meals per day in care** (median, IQR)	3	3–4	3	3–4	3	3–4
**Educator sample characteristics**	***n*** **51**		***n*** **69**		***n*** **120**	
**Age (years)** (median, IQR)†	39	32–49	35	28–47	37	29–48
**Gender** ‡						
Women/female	50	100	63	91	113	95
Self-ascribed	0	0	1	1	1	<1
**Highest qualification beyond secondary school** §						
None	2	4	2	3	4	3
Certificate	9	18	17	25	26	22
Diploma	26	51	31	45	57	48
Degree	13	26	19	27	32	27
**Experience** (median, IQR)						
Total time worked in childcare settings (years)	7·8	3·6–12·3	8·0	3·0–15·0	8·0	3·0–14·0

IQR, interquartile range, SEIFA, socio-economic indexes for areas (as per ABS classification of postcode ranking in Australia)^(30)^. Missing data:

* *n* 2.

† *n* 3.

‡ *n* 1.

§ *n* 1.

## Primary outcomes

### Effectiveness: children’s vegetable intake and variety

Vegetable intake data were collected from thirty-seven centres as two centres provided secondary outcome data but not primary outcome data. Nine centres provided data for ten or less children (*n* 5 control, *n* 4 intervention), and there was an average of twenty children (range: 1–57) per centre. At follow-up, children’s (*n* 719) usual vegetable intake over the past month was 0·98 (0·50–1·5) and 1·00 (0·50–1·75) serves per day in the control and intervention group, respectively. Nearly one in ten children (*n* 67, 9·3 %) did not consume any vegetables. Results of the two-part mixed model for children’s usual serves of vegetables consumed per day, adjusting for covariates, are shown in Table 2. There was no difference between the control and intervention group in the odds of children consuming any serves of vegetables compared with no vegetables (OR = 0·70 (95 % CI 0·34, 1·43)) (*P* = 0·32). Among those who consumed vegetables (*n* 652, >0 serves per day), children in the intervention group consumed 6 % more vegetable serves per day than those in the control group; however, the difference was not statistically significant (exp(b): 1·06 (95 % CI 0·78, 1·43)) (*P* = 0·71). The average serves of vegetables consumed per day among vegetable consumers were 1·40 (95 % CI 1·08–1·72) in the control group and 1·51 (95 % CI 1·20–1·82) in the intervention group (difference=0·11 serves per day).

**Table 2:** Two-part mixed models for children’s usual serves per day of vegetable intake (*n* 719) and vegetable variety (*n* 689) at follow-up

Outcome: usual vegetable intake	**Fixed effect:** population-level effect				**Random effect:** centre-level effect		
	Control	Intervention	95 % CI	***P*** **value**	Variance (ICC)	95 % CI	**LR test (** ***P*** **-value)**
**Part 1: logistic model (** ***n*** **719)**							
OR (95 % CI)	1 (ref)	0·70	0·34–1·43	0·325	0·33	0·09	0·015
**Part 2: gamma model (** ***n*** **652)** *							
exp(coefficient) (95 % CI)	1 (ref)	1·06	0·78–1·43	0·713	0·17	0·35	<0·001
**Outcome: vegetable variety**							
**Part 1: logistic model (** ***n*** **689)** †							
OR (95 % CI)	1 (ref)	0·73	0·40–1·32	0·293	0·13	0·04	0·138
**Part 2: Poisson model (** ***n*** **613)** ‡							
exp(coefficient) (95 % CI)	1 (ref)	1·07	0·88–1·32	0·485	0·07	0·34	<0·001

ICC, intraclass correlation coefficient; LR, likelihood ratio; Ref, reference category.

All models are adjusted for co-variates of child age, gender, centre size and socio-economic index for areas (SEIFA) ranking^(30)^.

* Mixed gamma model excludes children who consumed zero vegetables (*n* 67).

† Zero-inflated model excludes missing data (*n* 30).

‡ Mixed Poisson model excludes children with zero variety (*n* 76). LR test was performed to test mixed model *v*. simple model.

At follow-up, the variety of vegetables consumed by children (*n* 689) in the two most recent days in LDC after excluding missing data (*n* 30) was 3 (2–5) types of vegetables in the control group and 4 (2–5) types of vegetables in the intervention group. Around 11 % (*n* 76) of children were reported to consume zero types of vegetables in the two recent days in LDC. Results from the two-part zero-inflated mixed Poisson model for children’s vegetable variety, after adjusting for covariates, are shown in Table 2. Children in the intervention group were no more likely than children in the control group to consume one or more types of vegetables compared with none (*P* = 0·29). Among children that consumed one or more vegetable types (*n* 613), those in the intervention group consumed 7 % more types of vegetables (exp(b): 1·07 (95 % CI: 0·88, 1·32)) than those in the control group; however, the results were not statistically significant (*P* = 0·48). Among vegetable consumers, the average variety of vegetables consumed per day was 3·80 (95 % CI 3·23–4·37) in the control group and 4·11 (95 % CI 3·55–4·67) in the intervention group. The results of all models, including covariates, are available in see online supplementary material, Supplementary Table 1.

## Secondary outcomes

### Effectiveness: educator knowledge and skills

**Fig. 2:**
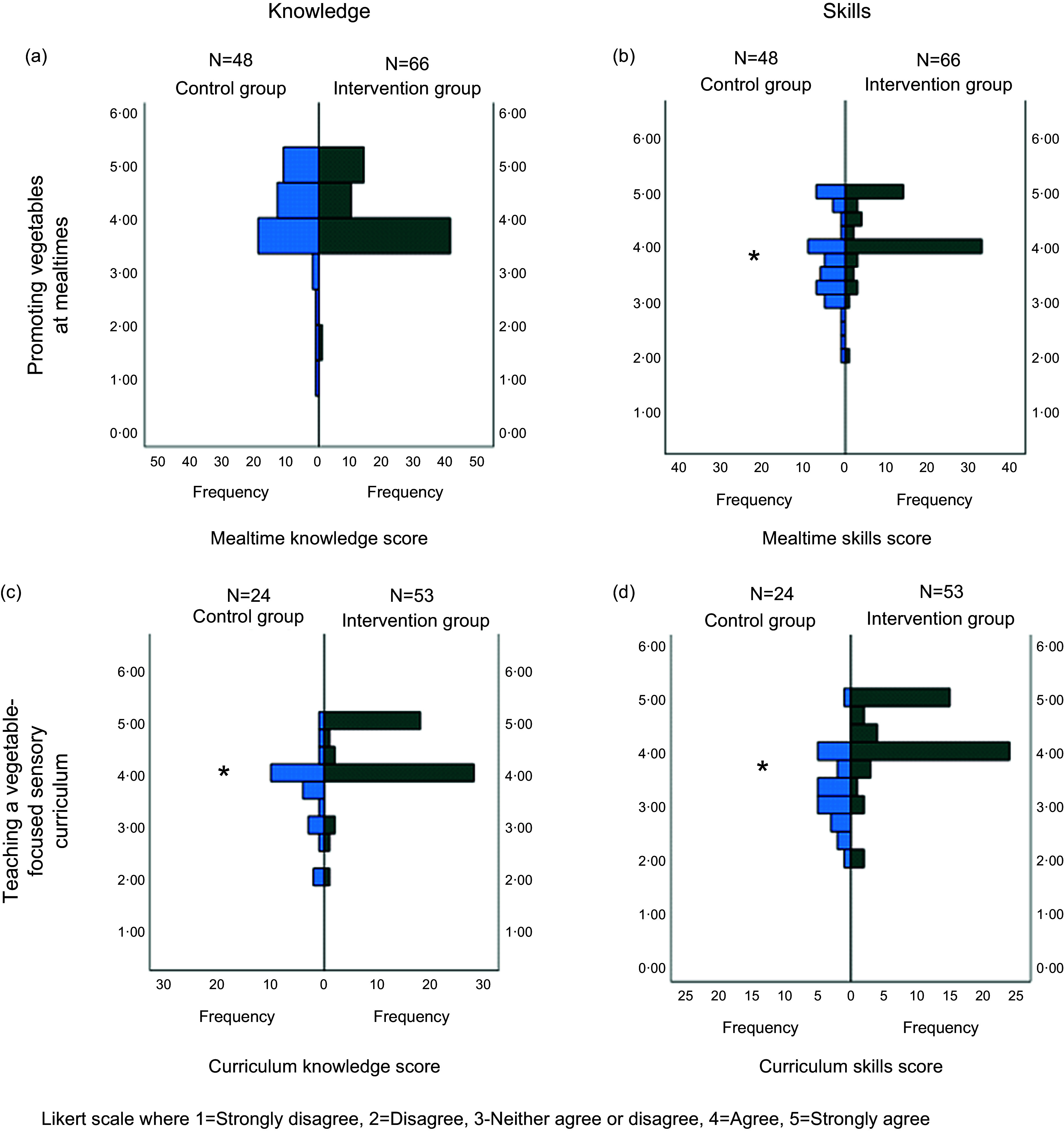
Frequency of educator’s theoretical domain framework questionnaire scores (Likert score responses 1–5) for knowledge and skills for promoting vegetables at mealtimes and teaching a vegetable focused sensory questionnaire for the control and intervention group at follow-up *P < 0·001

The distribution of educators’ scores at follow-up for the TDF domains of knowledge and skills for promoting vegetables at mealtimes and teaching a vegetable-focused sensory curriculum are displayed in Fig. 2. There were no statistically significant differences between the control (median (IQR)) (4·2 (4·0–4·7)) and intervention (4·0 (4·0–4·7)) groups (*P* = 0·79) for knowledge to promote vegetables at mealtimes (Fig. 2(a)). Skills in promoting vegetables at mealtimes were significantly higher (*P* < 0·001) in the intervention group (4·0 (4·0–4·8)) than in the control group (3·8 (3·3–4·4)) (Fig. 2(b)). Knowledge (Fig. 2(c)) and skills (b) in teaching a vegetable-focused sensory curriculum were significantly higher (both *P* < 0·001) in the intervention group (knowledge, 4·0 (4·0–5·0); skills, 4·0 (4·0–5·0)) than the control group (knowledge, 4·0 (3·1–4·0); skills, 3·3 (2·8–3·9)). Educators responses to the TDF statements are available in see online supplementary material, Supplementary Table 2.

### Implementation: intervention fidelity

Intervention fidelity was reported by twenty centres. Most (*n* 16/20) centres reported using the Mealtime environment initiative, and most educators (*n* 55/66) reported completing the online training (*n* 5/66 partially complete). Most centres reported delivering the Curriculum lessons (*n* 15/16 centres) and snack time activities (*n* 14/16 centres) (incomplete questionnaires for *n* 4 centres). Most educators in the intervention group reported teaching *most* (*9–15 lessons*) or *all (16 lessons*) lessons (*n* 38/53) and *most* (*9–15 activities*) or *all (16 activities*) snack time activities (*n* 35/53). Few educators did not teach any lessons (*n* 1/53) or snack time activities (*n* 3/53).

### Implementation: intervention acceptability

Acceptability for the Mealtime environment and Curriculum initiatives are shown in see online supplementary material, Supplementary Table 3. For educators who partially or fully completed the Mealtime environment training, there was strong agreement that the training provided them with practical strategies to promote vegetables (*n* 57/60) and that the strategies improved children’s liking of vegetables (*n* 50/60). For the educators who delivered the Curriculum, almost all agreed (*n* 49/51) that the curriculum helped children taste new vegetables.

### Reach and adoption

The intervention reached 307 centres of a total of 8556 LDC centres in Australia^(38)^ and was adopted by 50 of 226 (22 %) eligible centres who expressed interest in participating. Reasons for non-participation were obtained from eighteen centres and included: lack of staff resourcing, staff/director on leave or left role, lack of time or unable to participate (reasons not provided). There were no significant differences in socio-economic position (high/low SEIFA) (*P* = 0·644) and state representation (*P* = 0·107) for the centres that did participate (*n* 50) compared with centres that did not participate (*n* 176).

Overall, the study sample (*n* 39) had a higher proportion of centres from the state of New South Wales (21 of 39, 54 %) and Australian capital territory (3 of 39, 8 %) compared with the national representation of LDC in Australia which represent 39 % and 2 % of LDC centres respectively^(38)^. There was a lower proportion of centres from Victoria (4 of 39, 10 % compared with 21 %), Queensland (5 of 39, 13 % compared with 20 %) and South Australia (1 of 39, 3 % compared with 5 %)^(38)^. There were no centres from the state of Tasmania, which represents 1 % of LDC centres in Australia^(38)^.

### Contamination

There were eight centres from the intervention group and four centres from the control group who reported using Munch and Move resources for staff training and or/curriculum. All of these centres reported using the Munch and Move policy/resources at baseline in the registration questionnaire.

## Discussion

This pragmatic cluster randomised controlled trial, the third phase in the multiphase optimisation strategy (MOST) cycle, applied the RE-AIM framework to evaluate a 12-week intervention targeting the Mealtime environment and Curriculum for increasing children’s vegetable intake in LDC and understand its potential to be upscaled into LDC settings nationally^(21)^. While educators’ skills to promote vegetables at mealtimes and knowledge and skills to teach a vegetable-focused sensory curriculum increased, there were no statistically significant differences between the groups for usual vegetable intake, vegetable variety or the likelihood of children consuming at least some vegetables. Nonetheless, the small difference in usual vegetable consumption amongst vegetable consumers in the intervention group (equivalent to 0·11 serves of vegetables per day) was in the hypothesised direction. Further, reach of the intervention using real-world dissemination strategies was national (centres, educators and children in all states and territories in Australia other than Tasmania) and implementation of the self-delivered online intervention, replicating real-world conditions, was considered acceptable to educators and feasible in the LDC setting.

Identified in phase 2 of the MOST framework (i.e. the optimisation phase) as the initiative combination with the most promise for increasing children’s vegetable intake, the Curriculum with Mealtime environment intervention package was hypothesised in this evaluation phase study to be effective at increasing children’s vegetable intake. However, when evaluated in a 12-week randomised controlled trial, the effect on vegetable intake (0·11 serve per day, *P* = 0·71) was not statistically significant and was less than that observed in the previous optimisation phase study (0·36 serve per day, *P* = 0·06)^(20)^. This difference may be due to the different measures of vegetable intake used by the two studies, whereby this study used an educator-completed SFS-ECEC that relies on educators memory recall for multiple children, compared with the optimisation phase study which precisely measured intake using the weighed plate waste method^(20)^. The effect was also less than that observed in a 6-month theory-based multi-strategy intervention targeting Australian LDC centre food provision which saw a significant increase in children’s vegetable intake (also measured using the SFS-ECEC) of 0·4 serves per day (*P* < 0·001) compared with usual practice^(22)^. This study used multiple strategies for the implementation intervention such as securing executive support via face-to-face meetings, provision of group training and resources, audit and feedback via a dietitian, and one-on-one support by an experienced implementation officer. This suggests that the use of multiple strategies can lead to greater increases in vegetable intake, supported by a recent umbrella review^(13)^. However, the resource-intensive nature of such an approach limits scalability. Our study tested a more simplified and pragmatic approach using a single strategy intervention delivery approach, designed to replicate real-world conditions and improve scalability, in which centres were provided with intervention materials online for self-delivery. Thus, the 6 % non-significant increase (0·11 serves per day) in vegetable consumption demonstrates the potential for this real-world intervention approach to deliver a meaningful effect on children’s vegetable intake; however, further work is required to confirm this prior to scale up^(40,41)^.

Further to this, despite the non-significant impact on vegetable intake, educator’s knowledge and skills, as measured by the TDF, were significantly higher in the intervention group than in the control group post-intervention for teaching a vegetable-focused sensory curriculum and for promoting vegetables at mealtimes (skills only). These findings from our self-delivered intervention are notably better than previous researcher implemented multi-strategy interventions targeting food provision/service in the LDC setting. For example, two Australian studies found no significant differences between the control and intervention group for cooks’ knowledge and skills post-intervention^(34,36)^. Interestingly, Seward et al. (2018)^(34)^ found a 0·7 serve per day (*P* < 0·001) increase in children’s vegetable intake following a 6-month multi-strategy implementation intervention targeting centre food provision without increasing cook’s knowledge and skills. This suggests that the mechanism of action for improving children’s vegetable intake was through other pathways. Therefore, consideration of the role differentiation between cooks and educators is important due to the different pathways for influencing children’s intake. That is, cooks can influence what food is provided, whereas educators can influence what children consume from what is provided using strategies such as role modelling, positive reinforcement, encouragement and interactive educational activities^(13)^. Although there are limited studies investigating the impact of LDC interventions on TDF domains of knowledge and skills for educators and cooks, findings from these studies suggest that improving knowledge and skills may not necessarily translate to improvements in children’s dietary intake in the short term. Nonetheless, scalable interventions that increase educators’ knowledge and skills in vegetable-related practices and/or teaching are valuable, and it is possible that continued intervention implementation and thus greater exposure for children to evidence-based practices^(28)^ may lead to increases in children’s usual vegetable intake.

The pragmatic online self-delivered intervention approach, including real-world dissemination strategies used in the present study, was shown to be feasible and acceptable for adoption by Australian LDC centres and educators nationally. These findings are consistent with the previous optimisation phase that found good acceptability from educators and appropriate fidelity, confirming the feasibility of the self-delivered online intervention via a larger national trial. The use of digital platforms has recently been identified as an ideal delivery medium for public health nutrition interventions in the ECEC setting^(42)^, particularly given the COVID-19 pandemic and associated move towards online technologies, with an increase in the use of digital interventions to promote healthy eating in children seen in recent years^(43–45)^. In addition, interventions that are delivered online can have a greater reach in the community, negating the requirements for face-to-face delivery and usually at a lower cost. The feasibility of online intervention delivery in the ECEC setting has previously been recognised. For example, a recent intervention in LDC centres delivered an online web-based menu-planning tool and found good acceptability with variable engagement across the 12-month intervention^(46)^. Another pilot study in ECEC settings delivered an online nutrition support program (GO NAPSACC), which was effectively implemented by centre directors^(47)^. Further research exploring the barriers and enablers to adoption of digital interventions in ECEC is warranted.

Although this evaluation phase study confirmed feasibility of the intervention targeting the Mealtime environment and Curriculum, the vegetable intake findings did not confirm the effectiveness of the intervention in its current form. There were no significant differences between groups for vegetable intake or number of types of vegetables consumed, or the likelihood to consume some vegetables. Adaptations to improve the present intervention are required to further optimise intervention effectiveness in regard to vegetable intake and variety and impacting non-vegetable consumers. This can be done using the MOST cyclic approach through additional iterations of the preparation–optimisation–evaluation cycle, named the continuous optimisation principle^(48)^. This may involve returning to the preparation phase in which the importance of additionally targeting food provision was recognised given the influence cooks can have on the provision of vegetables to children and thus vegetable intake, while addressing barriers to implementation, e.g. insufficient time for LDC cooks identified in the previous optimisation phase. Future interventions can consider alternate approaches to better tailor interventions to centre needs by individualising intervention components and implementation strategies following baseline audits such as menu assessments (i.e. only centres with an identified need would adopt the intervention component for food provision).

A major strength of the present study is the application of the MOST^(18)^ and RE-AIM frameworks^(21)^. The MOST framework allowed for the optimal intervention from the previous optimisation phase study^(20)^ to be evaluated using the gold standard randomised controlled trial^(18)^. The RE-AIM framework^(21)^ was applied to evaluate the intervention and strengthen the translation potential from research into practice, employing a pragmatic approach to replicate real-world conditions through dissemination and implementation of a self-delivered online intervention. However, the maintenance domain of the RE-AIM framework was not assessed in the present study due to being out of scope.

Nonetheless, the short-term intervention duration (8-week active intervention period) was substantially less than the duration recommended for this setting^(13)^, and therefore may not have been long enough to see meaningful improvements in children’s usual vegetable intake. In addition, vegetable intake was reported by educators immediately post-intervention for children’s intake over the previous month, a period which may not have represented the full intervention effect. Further limitations include the lack of adjustment for baseline vegetable intake (not collected for pragmatic reasons described earlier) and the use of the modified SFS-ECEC, which has not been tested for reliability or validity. In addition, the method for collecting vegetable variety has not been validated and may not representative of usual variety as data was captured for the two most recent days in care.

The use of online self-completed data collection may have contributed to high levels of centres withdrawing or lost-to-follow-up (*n* 6 intervention centres, *n* 5 control centres). This study took place during the COVID-19 pandemic, impacting recruitment and staff capacity resulting in amendments to data collection procedures, whereby some centres completed surveys for a sub-sample of children rather than all children in attendance on one day. Therefore, it is possible that the selection of children may have been influenced by educator bias such as choosing children who like vegetables which may not be representative of the sample. In addition, the self-completed data collection may have resulted in inflated fidelity results, as educators who were more involved in the study (i.e. those who completed the training and taught the curriculum) may have been more likely to complete the questionnaires than those who did not complete the intervention. Further, food provision at the centre level was not measured and therefore cannot be used to understand whether the environment allowed for increases in intake, whilst blinding of educators reporting on the primary outcome of children’s vegetable intake was not possible. The results may not be generalisable as the study sample included only private LDC centres and contamination was possible given other initiatives (e.g. Munch and Move) may have already been part of centres usual practice prior to commencing the study and baseline data was not included in the analysis.

In conclusion, this study targeting the Mealtime environment and Curriculum in Australian LDC centres was designed and implemented to replicate real-world intervention delivery and thus improve translation potential. Although there was no statistically significant or meaningful effect on children’s usual vegetable intake while in care, the effect of the pragmatic self-delivered online intervention was in the expected direction with children in the intervention group consuming more vegetables than control children. In addition, the intervention had a positive effect on educator’s knowledge and skills and was considered to be feasible and acceptable, reaching centres, educators and children nationally. This demonstrates the potential of this intervention approach to be scalable and deliver a meaningful effect on vegetable intake in the future. However, further improvements to optimise the intervention are recommended and could occur via additional iterations using the MOST cyclic approach. This study contributes to research focusing on pragmatic interventions to improve children’s vegetable intake in ECEC settings, whereby future studies should consider real-world delivery approaches into the intervention design, important for informing policy and practice.

## Supporting information

Morgillo et al. supplementary material 1 Morgillo et al. supplementary material

Morgillo et al. supplementary material 2 Morgillo et al. supplementary material

## Supplementary material

For supplementary material accompanying this paper visit https://doi.org/10.1017/S1368980024000557
