# Supplementary material for: Evaluation of a long day care intervention targeting the mealtime environment and curriculum to increase children’s vegetable intake: a cluster randomised controlled trial using the multiphase optimisation strategy framework
Source: Public Health Nutr. 2024 Feb 26;27(1):e87. doi: 10.1017/S1368980024000557 (PMC10966837; doi:10.1017/S1368980024000557)
Supplement: Morgillo et al. supplementary material 1 — Morgillo et al. supplementary material [file S1368980024000557sup001.docx]

| **Supplementary table 1:** Two-part mixed effect models for children’s usual vegetable intake and variety with all co-variates at follow up | | | | | | |
| --- | --- | --- | --- | --- | --- | --- |
| Zero-inflated two-part mixed effect model |  | | | | | |
|  | Part 1: Logistic model (n = 719) | | | Part 2: Gamma model (n = 652)^*^ | | |
|  | **Odds ratio** | **95% CI** | **p-value** | **exp (coefficient)** | **95% CI** | **p-value** |
| **Outcome: Usual Vegetable Intake**  **Fixed effect:** Population level effect |  |  |  |  |  |  |
| Control | 1 (ref) |  |  | 1(ref) |  |  |
| Intervention | 0.70 | 0.34 – 1.43 | 0.325 | 1.06 | 0.78-1.43 | 0.713 |
| Age | 1.02 | 0.99-1.04 | 0.228 | 1.00 | 1.00-1.01 | 0.075 |
| Gender |  |  |  |  |  |  |
| Male | 1 (ref) |  |  | 1 |  |  |
| Female | 1.77 | 1.03-3.04 | 0.039 | 1.88 | 0.86-1.05 | 0.336 |
| Not specified | 1 (empty) |  |  | 3.41 | 0.94-12.37 | 0.062 |
| Centre size |  |  |  |  |  |  |
| Small (<50 places) | 1 (ref) |  |  | 1 (ref) |  |  |
| Large (>50 places) | 0.93 | 0.46-1.87 | 0.835 | 0.78 | 0.61-1.12 | 0.222 |
| SEIFA |  |  |  |  |  |  |
| Low (1-5) | 1 |  |  | 1 (ref) |  |  |
| High (6-10) | 0.63 | 0.31-1.25 | 0.186 | 0.72 | 0.77-1.40 | 0.820 |
| Constant | 8.1 | 2.03-32.25 | 0.003 | 10.60 | 0.78-1.74 | 0.453 |
|  | Part 1: Logistic Model (n = 689)^†^ | | | Part 2: Poisson Model (n = 613)^‡^ | | |
|  | **Odds ratio** | **95%CI** | **p-value** | **exp (coefficient)** | **95%CI** | **p-value** |
| **Outcome: Vegetable Variety** |  |  |  |  |  |  |
| **Fixed effect:** Population level effect |  |  |  |  |  |  |
| Control | 1 (ref) |  |  |  |  |  |
| Intervention | 0.73 | 0.40-1.32 | 0.293 | 1.07 | 0.88-1.32 | 0.485 |
| Age | 1.00 | 0.98-1.02 | 0.11 | 1.00 | 0.88-1.31 | 0.485 |
| Gender |  |  |  |  |  |  |
| Male | 1 (ref) |  |  | 1 (ref) |  |  |
| Female | 1.88 | 1.13-3.14 | 0.015 | 1.03 | 0.95-1.12 | 0.455 |
| Not specified | 1 (empty) |  |  | 0.90 | 0.28-1.89 | 0.866 |
| Centre size |  |  |  |  |  |  |
| Small (<50 places) | 1 (ref) |  |  | 1 |  |  |
| Large (>50 places) | 0.78 | 0.43-1.39 | 0.396 | 0.87 | 0.71-1.06 | 0.18 |
| SEIFA |  |  |  |  |  |  |
| Low (1-5) | 1 (ref) |  |  | 1 (ref) |  |  |
| High (6-10) | 0.72 |  |  | 1.06 | 0.87-1.30 | 0.553 |
| Constant |  | 2.98-37.78 | 0.00 | 3.47 | 2.59-4.65 | 0.00 |
| Ref=reference category. ^*^Gamma model excludes children who consumed zero vegetables (n=67). ^†^Zero-inflated Poisson model excludes missing data (n=30). ^‡^Poisson model excludes children with zero variety (n = 76). | | | | | | |

| **Supplementary table 2.** Educator responses (N) with agreement against the TDF statements for knowledge and skills for promoting vegetables at mealtimes and teaching a vegetable-focused sensory curriculum | | | | | | | | | | |  |
| --- | --- | --- | --- | --- | --- | --- | --- | --- | --- | --- | --- |
| **TDF Domain** | **Statement** | **Strongly disagree** | | **Disagree** | | **Neither agree or disagree** | | **Agree** | | **Strongly agree** | |
|  | **Promoting vegetables at mealtimes** | **Control**  (n=48) | **Int.**  (n=66) | **Control**  (n=48) | **Int.**  (n=66) | **Control**  (n=48) | **Int.**  (n=66) | **Control**  (n=48) | **Int.**  (n=66) | **Control**  (n=48) | **Int.**  (n=66) |
| **Knowledge** | I know what my responsibilities are with regard to promoting vegetables at mealtimes | 2 | 0 | 0 | 1 | 1 | 0 | 17 | 41 | 28 | 24 |
|  | I know how to promote vegetables at mealtimes | 1 | 0 | 2 | 1 | 2 | 0 | 23 | 44 | 20 | 21 |
|  | I know how to overcome barriers to promoting vegetables at mealtimes | 1 | 0 | 6 | 2 | 8 | 6 | 21 | 43 | 12 | 15 |
|  | I know when to apply strategies from the training when promoting vegetables at mealtimes | - | 0 | - | 1 | - | 3 | - | 47 | - | 15 |
|  | I am aware of the goals of the training | - | 0 | - | 1 | - | 2 | - | 40 | - | 23 |
|  | I am aware of the content of the training | - | 0 | - | 1 | - | 1 | - | 45 | - | 19 |
| **Skills** | I have received training in how to promote vegetables at mealtimes | 6 | 0 | 12 | 2 | 10 | 5 | 12 | 42 | 8 | 17 |
|  | I have the skills to promote vegetables at mealtimes | 0 | 0 | 5 | 1 | 8 | 6 | 23 | 38 | 12 | 21 |
|  | I have the skills to overcome barriers to promoting vegetables at mealtimes | 0 | 0 | 3 | 1 | 14 | 7 | 20 | 39 | 11 | 19 |
|  | I have practiced promoting vegetables at mealtimes | 0 | 0 | 1 | 1 | 0 | 1 | 30 | 45 | 17 | 19 |
|  |  | **Strongly disagree** | | **Disagree** | | **Neither agree or disagree** | | **Agree** | | **Strongly agree** | |
|  | **Vegetable focused-sensory curriculum** | **Control**  (n=24) | **Int.**  (n=53) | **Control**  (n=24) | **Int.**  (n = 53) | **Control**  (n = 24) | **Int.**  (n = 53) | **Control**  (n = 24) | **Int.**  (n = 53) | **Control**  (n = 24) | **Int.**  (n = 53) |
| **Knowledge** | I know what my responsibilities are with regard to teaching a vegetable-focused sensory curriculum | 0 | 0 | 2 | 1 | 4 | 2 | 14 | 31 | 4 | 19 |
|  | I know how to plan learning activities according to a vegetable-focused sensory curriculum | 0 | 0 | 3 | 2 | 6 | 2 | 13 | 30 | 2 | 19 |
|  | I know how to teach lessons and activities according to a vegetable-focused sensory curriculum | 0 | 0 | 3 | 2 | 7 | 2 | 12 | 29 | 2 | 20 |
|  | I am aware of the goals of the Curriculum | - | 0 | - | 1 | - | 3 | - | 30 | - | 19 |
|  | I am aware of the content of the Curriculum | - | 0 | - | 1 | - | 2 | - | 31 | - | 19 |
| **Skills** | I have received training/instructions regarding how to teach a vegetable-focused sensory curriculum | 4 | 0 | 8 | 2 | 5 | 4 | 6 | 30 | 1 | 17 |
|  | I have the skills needed to teach a vegetable-focused sensory curriculum | 0 | 0 | 2 | 2 | 6 | 3 | 14 | 29 | 2 | 19 |
|  | I have been able to put into practice teaching a vegetable-focused sensory curriculum | 0 | 0 | 4 | 2 | 5 | 4 | 14 | 30 | 1 | 17 |
| Int., Intervention group. TDF, Theoretical Domains Framework. Questions were adapted from Seward et al., 2017 TDF questionnaire developed for childcare cooks. | | | | | | | | | | |  |

| **Supplementary table 3**: Acceptability of the Mealtime environment (n = 60) and Curriculum (n = 51) initiatives: Number of educators in the intervention group who agree or strongly agree with the following LORI framework statements | |
| --- | --- |
| **LORI Statement** | **N agree/strongly agree** |
| **Mealtime Environment** | **N = 60** |
| **Content quality** |  |
| The level of detail in the training was appropriate | 57 |
| The training was useful | 54 |
| **Learning goal alignment** |  |
| The training provided me with practical strategies to promote vegetables at mealtimes | 57 |
| Using the strategies promoted in the training has improved children’s liking of vegetables | 50 |
| Using the strategies promoted in the training has helped children to taste new vegetables | 58 |
| **Motivation** |  |
| I found the training interesting | 53 |
| The training motivated me to promote vegetables at mealtimes | 53 |
| **Re-usability /accessibility** |  |
| The training is suitable for educators with different levels of experience | 55 |
| The training is suitable for educators working in different childcare centre environments | 56 |
| I would recommend this training to other educators | 54 |
| **Duration** |  |
| The duration or the training was appropriate | 54 |
| **Curriculum** | **N = 51** |
| **Learning goal alignment** |  |
| In my view, teaching the curriculum is worthwhile | 49 |
| Teaching the curriculum improved children’s liking of vegetables | 40 |
| The curriculum helped children to taste new vegetables | 49 |
| **Motivation** |  |
| I found the Curriculum interesting | 46 |
| The Curriculum was engaging for children | 44 |
| **Re-usability /accessibility** |  |
| The Curriculum is suitable for educators working in different childcare centres/environments | 46 |
| The Curriculum is suitable for children from different backgrounds | 49 |
| The Curriculum is suitable for children of different ages | 47 |
| I would recommend the Curriculum to other educators | 47 |
| **Duration** |  |
| The amount of time for the preparation of the curriculum was reasonable | 45 |
| LORI, Learning object review instrument | |
