# Supplementary material for: Evaluation of a long day care intervention targeting the mealtime environment and curriculum to increase children’s vegetable intake: a cluster randomised controlled trial using the multiphase optimisation strategy framework
Source: Public Health Nutr. 2024 Feb 26;27(1):e87. doi: 10.1017/S1368980024000557 (PMC10966837; doi:10.1017/S1368980024000557)
Supplement: Morgillo et al. supplementary material 2 — Morgillo et al. supplementary material [file S1368980024000557sup002.pdf]

Educator Questionnaire

Thank you for taking part in the VegKit long day care study to Evaluate a multi-component initiative package to increase children's vegetable intake. Participation in the questionnaire is voluntary.

This questionnaire is about your experience of taking part in the Mealtime environment initiative of the VegKit Long Day Care study.

If you have been involved in teaching the Taste and Learn Curriculum there will be additional questions about your experience

The questionnaire will take about 10-15 minutes to complete. If you didn't complete the initiatives, you can still complete the questionnaire as your feedback will still be valuable. Please answer the questions with your honest thoughts and opinions - there are no right or wrong answers!

Did you complete the 'Encouraging healthy eating in long day care' training for educators?

- ◯ Yes I completed the training
- ◯ No I started, but never completed
- ◯ No I never started

If no, please tell us why you did not complete the training?

____

Since you started the study have you undertaken any other nutrition or food related training or used any other nutrition or food related resources in your work?

- ◯ Yes
- ◯ No

If yes, what other resources/trainings have you used?

____

Please tell us how much you agree with the following statements about the 'Encouraging healthy eating in long day care' training for educators

	Strongly disagree	Disagree	Neither agree nor disagree	Agree	Strongly agree
The level of detail in the training was appropriate	◯	◯	◯	◯	◯
The training was useful	◯	◯	◯	◯	◯
The training provided me with practical strategies to promote vegetables at mealtimes	◯	◯	◯	◯	◯
Using the strategies promoted in the training has improved children's liking of vegetables	◯	◯	◯	◯	◯
Using the strategies promoted in the training has helped children to taste new vegetables	◯	◯	◯	◯	◯
I found the training interesting	◯	◯	◯	◯	◯

The training motivated me to promote vegetables at mealtimes	○	○	○	○	○
The training is suitable for educators with different levels of experience	○	○	○	○	○
The training is suitable for educators working in different childcare centre environments	○	○	○	○	○
I would recommend this training to other educators	○	○	○	○	○
The duration of the training was appropriate	○	○	○	○	○

PART 2 - YOUR VIEWS AND PRACTICES

In this section we would like to find out about your attitudes and practices related to using the information provided in the 'Encouraging healthy eating in long day care' training for educators

Please indicate to what extent you agree with the following statements about the 'Encouraging healthy eating in long day care' training for educators

	Strongly disagree	Disagree	Neither agree nor disagree	Agree	Strongly agree
I am aware of the goals of the training	○	○	○	○	○
I am aware of the content of the training	○	○	○	○	○
I know what my responsibilities are with regard to promoting vegetables at mealtimes according to the training	○	○	○	○	○
I know how to promote vegetables at mealtimes according to the training	○	○	○	○	○
I know how to overcome barriers to promoting vegetables at mealtimes according to the training	○	○	○	○	○
I know when to apply strategies from the training when promoting vegetables at mealtimes	○	○	○	○	○

I have received training in how to promote vegetables at mealtimes according to the training	○	○	○	○	○
I have the skills to promote vegetables at mealtimes according to the training	○	○	○	○	○
I have the skills to overcome barriers to promoting vegetables at mealtimes according to the training	○	○	○	○	○
I have practiced promoting vegetables mealtimes according to the training	○	○	○	○	○

How often do you do each of the following?

	Never	Rarely	Sometimes	Most of the time	Always
At mealtimes I encourage children to try vegetables	○	○	○	○	○
At mealtimes I talk with children about vegetables, in terms of their appearance, colour, texture, smell or taste	○	○	○	○	○
I model enjoying vegetables by eating vegetables with children at mealtimes	○	○	○	○	○
If a child doesn't want to eat a vegetable, I don't offer that vegetable the next time	○	○	○	○	○
I force children to eat vegetables even if they don't want to eat them	○	○	○	○	○

Please indicate to what extent you agree with the following statements about the 'Encouraging healthy eating in long day care' training for educators

	Strongly disagree	Disagree	Neither agree nor disagree	Agree	Strongly agree
Promoting vegetables at mealtimes according to the training is part of my role as an educator	○	○	○	○	○
It is my responsibility to promote vegetables at mealtimes according to the training	○	○	○	○	○

Promoting vegetables at mealtimes according to the training is consistent with other aspects of my job	◯	◯	◯	◯	◯

Please indicate to what extent you agree with the following statements about the 'Encouraging healthy eating in long day care' training for educators (one response per question).

	Strongly disagree	Disagree	Neither agree or disagree	Agree	Strongly agree
I intend to promote vegetables at mealtimes according to the training at every mealtime	◯	◯	◯	◯	◯
I will definitely promote vegetables at mealtimes according to the training at every mealtime	◯	◯	◯	◯	◯
I intend to continue to promote vegetables at mealtimes according to the training over the next three months	◯	◯	◯	◯	◯
I have a strong intention to promote vegetables at mealtimes according to the training at every mealtime	◯	◯	◯	◯	◯

Any comments or feedback about the VegKit mealtime training initiative?

____

Are you involved in teaching the curriculum in the room?
(e.g. did you participate in teaching the lessons or activities for the VegKit 'Taste & Learn' curriculum)

◯ Yes
◯ No

The following questions are about the 'Taste & Learn' curriculum

How many of the lessons (intentional activities) from the Taste & Learn™ Curriculum were taught in your room?

(The curriculum comprised of 16 lessons e.g. The five senses, Food adventurer, Plant parts, Lets get cooking, Science and food)

◯ None
◯ Some (1-7 lessons)
◯ Half (8 lessons)
◯ Most (9-15 lessons)
◯ All (16 lessons)

If you did not teach all the lessons in full, please tell us why not?

____

How many of the snack-time activities from the Taste & Learn™ Curriculum did you teach?<br><br>(There were 16 snack time activities, 2 per week e.g. carrot three ways)	◯ None<br>◯ Some (1-7 snack times)<br>◯ Half (8 snack times)<br>◯ Most (9-15 snack times)<br>◯ All (16 snack times)
If you did not teach all the snack-time activities in full, please tell us why not?	__________________________________
Did you use any of the additional resources or supporting activities?<br><br>e.g. Group, progress chart, Reading corner, Music, home corner, writing/drawing area, outdoor play, sensory table	◯ Yes<br>◯ No
Did you use the vegetables that were suggested for the snack time activities	◯ Yes<br>◯ No
Since you started participating in this study have you taught any other nutrition or vegetable related lessons or activities (not including the Taste & Learn™ (Early Years) curriculum)?	◯ Yes<br>◯ No
what other lessons or activities have you taught?	____________________________

Please tell us how much you agree with the following statements about the 'Taste & Learn™ (Early Years) Curriculum' (circle one response per question).

	Strongly disagree	Disagree	Neither agree nor disagree	Agree	Strongly agree
In my view, teaching the curriculum is worthwhile	◯	◯	◯	◯	◯
Teaching the Curriculum improved children's liking of vegetables	◯	◯	◯	◯	◯
The curriculum helped children to taste new vegetables	◯	◯	◯	◯	◯
I found the Curriculum interesting	◯	◯	◯	◯	◯
The Curriculum was engaging for children	◯	◯	◯	◯	◯
The Curriculum is suitable for educators working in different childcare centres/environments	◯	◯	◯	◯	◯
I would recommend the Curriculum to other educators	◯	◯	◯	◯	◯

The Curriculum is suitable for children from different backgrounds (including cultural, religious etc)	○	○	○	○	○
The Curriculum is suitable for children of different ages	○	○	○	○	○
The amount of preparation for the Curriculum was reasonable	○	○	○	○	○

In this section we would like to find out about your attitudes and practices related to using the Taste & Learn™

Please indicate to what extent you agree with the following statements about the Taste & Learn™ (Early Years) Curriculum (circle one response per question

	Strongly disagree	Disagree	Neither agree nor disagree	Agree	Strongly agree
I am aware of the goals of the Curriculum	○	○	○	○	○
I am aware of the content of the Curriculum	○	○	○	○	○
I know what my responsibilities are with regard to teaching the Curriculum	○	○	○	○	○
I know how to plan learning activities according to the Curriculum	○	○	○	○	○
I know how to teach lessons and activities according to the Curriculum	○	○	○	○	○
I have received instructions regarding how to teach the Curriculum	○	○	○	○	○
I have the skills needed to teach the Curriculum	○	○	○	○	○
I have been able to put into practice teaching the Curriculum	○	○	○	○	○

Please indicate to what extent you agree with the following statements about the Taste & Learn™ (Early Years) Curriculum (circle one response per question).

	Strongly disagree	Disagree	Neither agree nor disagree	Agree	Strongly agree
Teaching the Curriculum is part of my role as an educator	○	○	○	○	○

It is my responsibility as an educator to teach vegetable-focused learning activities according to the Curriculum	○	○	○	○	○
Teaching the Curriculum is consistent with other aspects of my job	○	○	○	○	○

Please indicate to what extent you agree with the following statements about the Taste & Learn™ (Early Years) Curriculum (circle one response per question).

	Strongly disagree	Disagree	Neither agree nor disagree	Agree	Strongly agree
I intend to teach the Curriculum every year	○	○	○	○	○
I will definitely teach the Curriculum every year	○	○	○	○	○
I have a strong intention to teach the Curriculum again	○	○	○	○	○

Do you have any comments/feedback about the VegKit Curriculum initiative?

____

Thank you for completing the questionnaire and for participating in the VegKIT long day care study
